# Cardiovascular disease risk factors in Spain: A comparison of native and immigrant populations

**DOI:** 10.1371/journal.pone.0242740

**Published:** 2020-11-30

**Authors:** Elena Rodriguez-Alvarez, Nerea Lanborena, Luisa N. Borrell

**Affiliations:** 1 Department of Nursing I, University of the Basque Country (UPV/EHU), Leioa, Spain; 2 OPIK-Research Group for Social Determinants of Health and Demographic Change; 3 Department of Epidemiology & Biostatistics, Graduate School of Public Health & Health Policy, City University of New York, New York, NY, United States of America; 4 Department of Surgery, Medical and Social Science, University of Alcalá, Madrid, Spain; Universidade Federal de Minas Gerais, BRAZIL

## Abstract

Cardiovascular disease (CDV) risk factors are highly prevalent among adults with low social class in Spain. However, little is known on how these factors are distributed in the immigrant population, a socio-economic disadvantaged population. Thus, this study aims to examine inequalities in CVD risk factors among immigrant and native populations. We conducted a cross-sectional study using data from the Spanish National Health Survey 2017 and used log-binomial regression to quantify the association of immigrant status on CVD risk factors among adults aged 25–64 years. The probabilities of having at least three CVD risk factors were higher for immigrants from Eastern Europe (PR: 1.25; 95% CI: 1.15–1.35) and lower for immigrants from Africa (PR: 0.79; 95% CI: 0.69–0.89) when compared with natives. The association of immigrant status and CVD risk factors varies with educational attainment (p-interaction = 0.001). Immigrants from Eastern Europe with low educational attainment have a higher probability of having at least three CVD risk factors compared with their native counterparts. In contrast, immigrants from Africa and Latin America with low educational attainment had a protective effect against having at least three CVD risk relative to natives. Health prevention and promotion strategies to reduce the burden of CVD taking should account for educational attainment given its differential effect among the immigrant population in Spain.

## Introduction

Cardiovascular diseases (CVD) is the leading cause of mortality worldwide, accounting for 31% of all deaths [1]. In addition, they represent an important cause of premature death (i.e., deaths before 65 years of age), disability and healthcare cost [2]. Although there has been a decrease in CVD-specific mortality in developed countries in recent decades [3], CVD continues to be the leading cause of death [4]. Moreover, the prevalence of CVD has increased, due to the aging of the population and the increase in the prevalence of risk factors [5]. In fact, in Europe, the prevalence of obesity and diabetes has doubled whereas the prevalence of hypertension and smoking have decreased in the last 30 years [3]. In addition, evidence suggests an unequal distribution of cardiovascular risk factors depending on income and educational level across countries [6]. The latter has led to higher mortality rates across European countries among lower socioeconomic groups [7, 8].

In the last two decades, the increase in migratory flows from low- to high-income countries has shaped the sociodemographic reality with implications for health and its policies [9]. In fact, place of birth constitutes a social determinant of health that generates health inequities for the immigrant population, due to their worse living and working conditions, less access to health services and their lower socioeconomic level [10]. In Europe, there is still little evidence on inequalities in cardiovascular risk factors. Studies show worse cardiovascular health in immigrants compared with natives [11, 12] and higher prevalence of hypertension, diabetes and obesity [13–15]. In addition, the prevalence of these risk factors varies according to place of birth [16, 17] and country of destination [18], as a consequence of regional differences in educational opportunities, income distribution, and/or access to health care [19].

Spain is currently the fourth country in the European Union when it comes to proportion of immigrant population (6,123,769) and has one of the highest proportions of immigrants relative to its total population (10.7% in 2019) [20]. The majority of immigrants come from Latin America (41%, mostly from Colombia, Ecuador, Venezuela, Peru and Bolivia), Maghreb (14%; mostly from Morocco), sub-Saharan Africa (4%; mostly from Senegal and Nigeria), Asia (7%; mostly from China) and Eastern Europe (16%; mostly from Romania, Bulgaria, Russia and Ukraine). However, only four studies have examined inequalities in cardiovascular health according to place of birth [21–24]. Two of these studies focused on inequalities in mortality [21, 22] and the other two examined specific CVD risk factors in two regions of Spain [23, 24] without consideration of socioeconomic position. Given that in Spain, CVD is the main cause of mortality [25], cardiovascular risk factors are more common in individual from low social class [26], and the high proportion of immigrants in the population, who are often of low socioeconomic position [20], this study aims to examine inequalities in CVD risk factors among immigrant and native populations using data from the 2017 Spanish National Health Survey; and whether these inequalities vary with educational attainment. We hypothesize that immigrants will have a higher prevalence of CVD risk factors than the native Spanish population.

## Materials and methods

### Study design and population

We conducted a cross-sectional design study among adults aged 25 to 64 using data from Spanish National Health Survey (SNHS) for 2017. The SNHS is a representative survey of the non-institutionalized Spanish population selected through a multi-stage stratified sampling. More detailed information on the methodology of this survey has been described elsewhere [27]. The study was based on information collected through a personal interview in selected households including 23,089 people, with a response rate of 74%. We excluded records of adults under 25 years of age (n = 1,645) because of their low prevalence of risk factors, lack of complete education and/or being unemployed. The latter makes it hard to assign a social class and employment status to those under 25 years of age. In addition, we excluded records of adults older than 65 years (n = 7,032) because they are less active among the native population and because the emigration unhealthy elderly immigrants tend to return to their home countries, a phenomenon refers to as “salmon bias” [28].

### Variables

Consistent with the American Heart Society (AHA) [29], the dependent variable was a CVD risk factors summary score including 3 health risk factors (diabetes, cholesterol and hypertension) and 4 health risk behaviors (overweight / obesity, smoking, poor diet and physical inactivity). These health and behaviors risk factors were self-reported. Hypertension, high cholesterol and diabetes were defined using the answer to the question (yes/no) of whether your doctor had ever told you that you had hypertension, diabetes and cholesterol? Smoking status (yes/no) was defined by aggregating those who smoked daily, occasionally, and those who had previously smoked in the same category as smokers [30] and those who reported as not smokers. Overweight/obesity, calculated using self-reported weight and height, was defined according to the World Health Organization [31] as BMI ≥ 25 kg/m^2^,. For diet, the Healthy Eating Index (EHI) for the Spanish population was used [32]. The EHI includes 10 indicators, with a range from 0 to 100 with a score of less than 80 considered as an unhealthy diet. Physical inactivity was determined from the International Physical Activity Questionnaire Short Form (IPAQ-SF) by measuring the time in minutes of physical activity during the last week. Physical inactivity was defined as either <75 minutes per week of vigorous intensity activity<150 minutes per week of moderate intensity activity or <150 minutes per week of moderate and vigorous intensity activity [30]. Consistent with a previous study [33, 34], a score was created by summing the number of CVD risk factors, with a range of 0 to 7. We then used the sample median distribution of the score, ≥3 CVD risk-factors, as cut point [34] to specify the outcome.

Country of birth, the independent variable, was categorized as natives for those born in Spain and immigrants for those born in a country with a Human Development Index (HDI) <0.80 [35]. We further categorized immigrants according to the region of origin in Eastern Europe (non-European Union, Romania and Bulgaria), Latin America and Africa. Please see Appendix 1 for a list the countries included in each region of origin. Hereafter, we referred to country of birth as immigrant status and to the Spanish population as natives.

Consistent with other studies [36, 37], we included as covariates age (as a continuous variable), sex, educational attainment (primary, secondary and university), living arrangement (as a couple, others), and employment status (working, in unemployment and others). In addition, we included occupational social class. Social class was based on the of the head of household (current, last or never) following the Goldthorpe-oriented classification proposed by the Spanish Society of Epidemiology [38]. Five groups were identified (I Managers with >10 workers; II Managers with <10 workers; III Intermediary and self-employed; IV Supervisors, qualified and semi-qualified; V Non-qualified workers). Occupational social class was further categorized as non-manual workers (class I, II and III) and manual workers (class IV and V).

Of the 14,421 people who completed the individual questionnaire, we excluded those born in countries with a very high HDI (>0.80; n = 363) because they have similar or better CVD profile and socioeconomic position than the Spanish population [39] and records without information for any of the cardiovascular risk factors: cholesterol (n = 4), smoking (n = 10), BMI (n = 355), diet (n = 479) and physical inactivity (n = 14). In addition, we exclude records of those without information on occupational social class (n = 154), employment status (n = 21) and marital status (n = 19). Immigrants from Asia were also excluded due to small sample size (n = 65). These exclusions resulted in an analytical sample of 12,937 including 1,271 immigrants. Please see Appendix 2 for a comparison of included and excluded records.

### Statistical analysis

Descriptive statistics for selected characteristics were calculated for the total population and by immigrant status. In addition, the prevalence estimates for CVD risk factors were calculated by immigrant status. Chi-squared of independence statistics were used to assess associations: 1) between each covariate and immigrant status, and 2) between the prevalence of CVD risk factors and immigrant status. Log binomial regression was used to quantify the association of immigrant status and cardiovascular risk factors before and after controlling for the selected covariates. In addition of educational attainment, we tested interactions terms of immigrant status with age and sex, in the fully-adjusted model because immigrants tend to be younger and more likely to be female [40] than the native population.

Data management procedures were carried out using SPSS 24.0. (IBM, Armonk, NY, USA) whereas the statistical analyses were conducted using SUDAAN 11.0.1 (RTI, Research Triangle Park, NC, USA) to take into account the complex sampling design and yield unbiased standard error estimates. Sample sizes presented in Tables 1 and 2 are unweighted. However, proportions, standard errors (SE), prevalence ratios (PR) and 95% confidence intervals (CI) are weighted.

**Table 1:** Distribution of selected characteristics for participants by region of origin: Spanish National Health Survey, 2017.

	Spain n = 11,666%(SE)	Eastern Europe n = 296%(SE)	Latin America n = 627%(SE)	Africa n = 348%(SE)	P-value*
***Age (years)***					<0.001
25–44	47.7 (0.5)	67.9 (3.0)	63.9 (2.2)	68.3 (3.0)	
45–64	52.3 (0.5)	32.1 (3.0)	36.1 (2.2)	31.7 (3.0)	
***Gender***					<0.001
Men	51.5 (0.5)	43.4 (3.3)	38.2 (2.3)	53.3 (3.2)	
Women	48.6 (0.5)	56.6 (3.3)	61.8 (2.3)	46.7 (3.2)	
***Educational attainment***					<0.001
Primary or less	44.7 (0.5)	30.2 (3.1)	32.9 (2.2)	69.0 (3.0)	
Secondary	31.3 (0.5)	55.9 (3.3)	47.1 (2.3)	22.4 (2.7)	
Graduate or higher	24.0 (0.4)	14.0 (2.3)	20.0 (1.8)	8.6 (1.8)	
***Occupational social class***					<0.001
Manual	56.9 (0.5)	85.0 (2.5)	75.7 (2.0)	88.7 (2.0)	
No manual	43.1 (0.5)	15.0 (2.5)	24.3 (2.0)	11.3 (2.0)	
***Employment status***					<0.001
Employed	68.6 (0.5)	65.8 (3.2)	69.1 (2.1)	53.7 (3.2)	
Unemployed	15.0 (0.4)	23.3 (2.8)	20.2 (1.8)	20.4 (2.4)	
Others	16.3 (0.4)	10.9 (2.1)	10.7 (1.4)	25.9 (2.9)	
***Living arrangement***					<0.001
Couple	66.8 (0.5)	70.5 (3.0)	61.8 (2.2)	77.0 (2.7)	
Other	32.2 (0.5)	29.5 (3.0)	38.2 (2.2)	23.0 (2.7)	
***CVD risk health factors***					<0.001 0.008 0.374
Hypertension	15.4 (0.4)	11.7 (2.2)	9.9 (1.4)	8.7 (1.9)	
High Cholesterol	16.3 (0.4)	12.9 (2.3)	12.7 (1.6)	10.0 (1.9)	
Diabetes	4.5 (0.2)	2.4 (1.0)	4.2 (0.9)	5.5 (1.6)	
***CVD risk health behaviors***					<0.001 0.221 <0.001 <0.001
Smoking	59.3 (0.5)	64.1 (3.2)	32.1 (2.1)	29.5 (2.9)	
Overweight/obesity	53.4 (0.5)	57.0 (3.3) 88.1	56.9 (2.3)	50.2 (3.2)	
Unhealthy diet	80.6 (0.4)	(2.1)	83.0 (1.7)	70.4 (3.0)	
Insufficient physical activity	65.3 (0.5)	76.4 (2.8)	66.8 (2.2)	82.5 (2.5)	

CVD, cardiovascular disease.

*P-value from Chi-squared statistics.

**Table 2:** Prevalence of cardiovascular disease risk factors according to region of origin: Spanish National Health Survey 2017.

	Spain n = 11,666%(SE)	Eastern Europe n = 296%(SE)	Latin America n = 627%(SE)	Africa n = 348%(SE)	P-value*
***N° CVD risk health factors*, *% (SE)***					<0.001
0	72.9 (0.5)	79.0 (2.7)	78.5 (1.9)	81.3 (2.6)	
1	19.5 (0.4)	16.1 (2.4)	16.5 (1.7)	14.1 (2.3)	
2	6.2 (0.3)	4.0 (1.5)	4.5 (1.0)	3.7 (1.3)	
3	1.4 (0.1)	0.9 (0.6)	0.5 (0.3)	0.9 (0.6)	
***N° CVD risk health behaviors*, *% (SE)***					<0.001
0	1.6 (0.1)	1.3 (0.7)	2.1 (0.6)	-	
1	12.3 (0.4)	5.1 (1.4)	13.6 (1.6)	16.0 (2.5)	
2	30.6 (0.5)	25.1 (2.9)	39.1 (2.3)	42.7 (3.2)	
3	36.9 (0.5)	43.7 (3.3)	33.6 (2.2)	34.2 (3.0)	
4	18.6 (0.4)	24.8 (2.9)	11.6 (1.4)	7.1 (1.6)	
***N° all-CVD risk factors*, *% (SE)***					<0.001
0	1.3 (0.1)	1.1 (0.7)	1.8 (0.6)	-	<0.001
1	10.3 (0.3)	5.1 (1.4)	11.3 (1.5)	14.6 (2.4)	
2	25.7 (0.5)	20.8 (2.8)	31.9 (2.2)	35.7 (3.1)	
3	31.6 (0.5)	37.0 (3.2)	34.5 (2.2)	33.0 (3.0)	
4	20.7 (0.4)	27.5 (2.9)	16.7 (1.7)	12.9 (2.1)	
5	7.2 (0.3)	6.8 (1.8)	2.7 (0.6)	3.0 (1.1)	
6	2.7 (0.2)	1.3 (0.6)	0.8 (0.4)	0.7 (0.5)	
7	0.4 (0.1)	0.5 (0.5)	0.3 (0.3)	0.2 (0.2)	
***CVD risk factors Summary*, *mean (SE)***	2.95 (0.014)	3.13 (0.075)	2.66 (0.051)	2.57 (0.068)	
***≥3 CVD risk-factors*, *% (SE)***	62.6 (0.5)	73.0 (3.0)	55.0 (2.3)	49.8 (3.2)	<0.001

CVD, cardiovascular disease.

*P-value for chi-squared statistics and ANOVA.

## Results

Table 1 shows the distribution of sociodemographic characteristics as well as CVD risk factors and health behaviors by immigrant status. Compared with Spanish native adults, immigrants were younger, with a higher proportion of women among those from Eastern Europe and Latin America but lower proportion of women among those from Africa, less educated. In addition, immigrants were more likely to be in the manual occupational social class, unemployed and lived with a partner, except for those from Latin America than natives (all p values <0.001). Regarding CVD risk health factors, immigrants reported lower prevalence of hypertension and cholesterol compared with natives (p-values <0.01). However, there were no differences in the prevalence of diabetes between natives and immigrant populations, although the highest prevalence of diabetes was observed among immigrants from Africa. Compared with natives, immigrants were less likely to be smokers (except among those from Europe), more likely to have an unhealthy diet (except among those from Africa) and more likely to be physically inactive (p-value <0.05). There was no difference in the prevalence of overweight/obesity between immigrants and natives.

The prevalence of CVD risk factors according to immigrant status is shown in Table 2. The mean CVD risk factor score was higher among immigrants from Eastern Europe ($\bar{x}$ = 3.1) and lower among those from Africa ($\bar{x}$ = 2.57) relative to Spanish natives (p-value <0.001). Compared with natives, immigrants from Eastern Europe had a higher prevalence of having at least three CVD risk factors, (73.0%) whereas those from Latin America (55.0%) and Africa (49.8%) had a lower prevalence estimates (p-value <0.001).

Table 3 presents the unadjusted and adjusted PRs and 95% CIs for CVD risk factors. When compared with natives, the probability of having at least three CVD risk factors 16% (PR1.16; 95%CI: 1.07, 1.26) greater among immigrants from Eastern Europe whereas the probabilities were 12% (PR: 0.88; 95% CI: 0.81, 0.95) and 21% (PR: 0.79; 95% CI: 0.70, 0.90) lower among immigrants from Latin America and Africa, respectively. After adjustment, the associations were significant among immigrants from Eastern Europe (PR: 1.25; 95% CI: 1.15, 1.35; Table 3) and from Africa (PR: 0.79; 95% CI: 0.69, 0.89) only. Appendix 3 presents the unadjusted and adjusted PRs and their 95% confidence intervals for region of origin for each CVD risk factor.

**Table 3:** Prevalence Ratios and their 95% confidence intervals for region of origin on the cardiovascular disease risk factors, Spanish National Health Survey 2017.

	Unadjusted	Adjusted *
***≥3 CVD risk-factors***		
**Spain**	1	1
**Eastern** Europe	1.16 (1.07–1.26)	1.25 (1.15–1.35)
Latin America	0.88 (0.81–0.95)	0.95 (0.87–1.03)
Africa	0.79 (0.70–0.90)	0.79 (0.69–0.89)

CVD, cardiovascular disease.

*Adjusted for age (continuous), sex, employment status, living arrangement, education attainment and

**occupational** social class.

**Fig 1:**
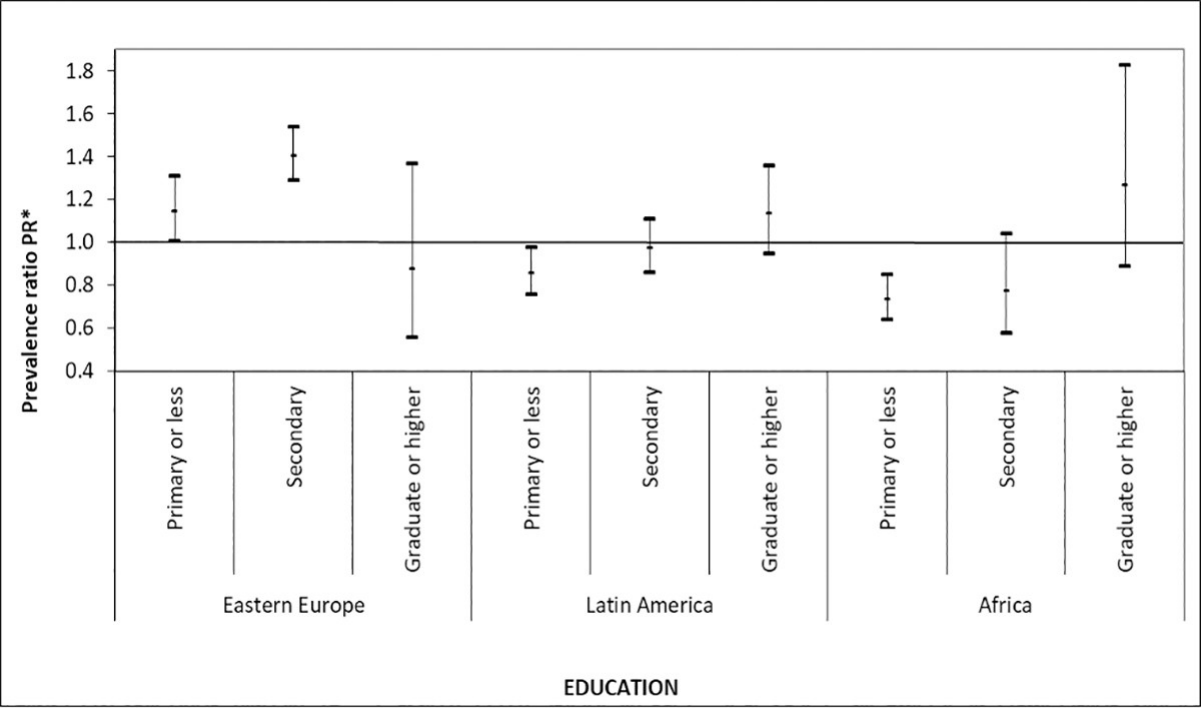
Prevalence Ratios (PR) and 95% CI confidence intervals for region of origin on ≥3 CVD risk factors by educational attainment: Spanish National Health Survey 2017. *Adjusted for age (continuous), sex, employment status and living arrangement.

Heterogeneity of the association between immigrant status and CVD risk factors was neither observed between immigrant status and age groups (p-interaction = 0.59) nor immigrant status and sex (p-interactions = 0.50). However, we observed heterogeneity in the association between immigrant status and CVD risk factors by educational attainment (p-interaction = 0.001). When compared with natives, the probability of having at least three CVD risk factors was greater in immigrants from Eastern Europe with primary and secondary educational attainment (PR: 1.15; 95% CI: 1.01, 1.31 and PR: 1.41; 95% CI: 1.29, 1.54, respectively), whereas the probability was lower among immigrants from Latin America (PR:0.86; 95% CI: 0.76, 0.98) and Africa (PR:0.74; 95% CI: 0.64, 0.85) with a primary educational attainment (Fig 1). It is worth noting that among immigrants from Latin American and African, the probability of having at least three CVD risk factors appears to increase with educational attainment.

## Discussion

This study showed inequalities in CVD risk factors between immigrants and natives in Spain. After adjustment for selected characteristics, we found a greater probability of having at least 3 CVD risk factors in immigrants from Eastern Europe and a lower probability among immigrants from Africa relative to Spanish natives. In addition, these associations varied with education attainment. Specifically, immigrants with less education from Eastern Europe were more likely to have at least three CVD risk factors compared with their Spanish native counterparts. However, in immigrants from Africa and Latin America, a primary level of education was protective against having at least three CVD risk factors relative to Spanish natives with the same educational attainment.

The differences in prevalence of CVD risk factors between the immigrant and native populations are largely dependent on place of birth [14, 15, 17, 23] type of CVD risk factor and host country of residence [16]. The latter is a consequence of regional differences and contextual factors including the degree of ethnic integration/acculturation, health beliefs and socioeconomic factors [18, 19]. Despite these differences, the evidence is consistent suggesting inequalities in CVD risk factors by region of birth. In our study, immigrants from Eastern Europe had a higher probability of having at least 3 CVD risk factors compared with natives. Related to our findings, Regidor et al. found higher CVD mortality rates among immigrants from Eastern Europe residing in Spain [21]. These high mortality rates observed among immigrants from Eastern Europe may be explained by the high prevalence of tobacco, overweight/obesity [41] and unhealthy diet [42] in their countries of origin. In fact, inequalities in CVD risk factors are related to increase risk of CVD morbidity and mortality [43]. In contrast, immigrants from Africa presented a better cardiovascular health profile, with a lower probability of having at least three CVD risk factors compared with natives. Limited evidence exists with regards to the changes in health behaviors such as diet, smoking habits and physical activity, in the host country for African immigrants. However, two studies in the Netherlands found lower prevalence of smoking [44] and a healthier diet among immigrants from Morocco, a group representing most of the African immigrant population in Spain, compared with their Dutch counterparts, regardless of socioeconomic position [45]. Thus, their lifestyle habits could explain the observed better cardiovascular profile in African immigrants in our study.

Despite the extensive evidence on the protective effect of high educational attainment on cardiovascular health [7, 8, 46, 47], this study shows that the effect of education differed among immigrants according to their regions of origin. Two previous studies, one in Norway [11] and another in the Netherlands [48] have examined the association of education with CVD risk factors, by place of birth. In both studies, educational attainment had a different effect for the immigrant and the native populations. These studies found that low educational attainment was associated with higher probability of CVD risk factors in immigrants compared to the native population with the exception of those from Morocco. Consistent with these findings, we observed that the probability of having at least three CVD risk factors was greater among immigrants from Eastern Europe with low educational attainment compared with natives. The opposite was true for immigrants from Africa and Latin America. Thus, high educational attainment may not carry health benefits for CVD risk in immigrants from Africa and Latin America. This differential effect may be related to structural discrimination in the labor market in Spain, which concentrates immigrants in the most precarious sectors regardless of their educational attainment, with little upward mobility. Specifically, immigrants from Africa have the greatest difficulties in accessing the labor market, with high unemployment rates and informal employment, and like immigrants from Latin America, high rates of job insecurity [49, 50]. Furthermore, this labor market is characterized by high levels of physical demand, low wages, and therefore, less access to resources and consumer goods. Hence, incorporation into the labor market requires immigrants to be in good health. These issues could explain the observed better cardiovascular profile associate with the lowest educational attainment among immigrants from Africa and Latin America.

This study is not without limitations. First, it is a cross-sectional study and does not allow us to make causal inferences or examine changes over time for CVD risk factors. Second, CVD risk factors were self-reported, which raises possible recall biases and social desirability. The biases if they were to occur, they may have been non-differential and could either over- or under-estimate our results toward the null. However, if we assume that immigrants face more barriers to access the health system, their probability of being diagnosed with hypertension, diabetes or high cholesterol levels may be lower than natives. The latter may lead to differential misclassification and a potential underestimation of the PRs we observed. Third, immigrants with lower levels of education and undocumented are less likely to participate in national and international survey [51]. However, while the overall response rate was 74% for the SNHS, country of origin was not a variable considered for stratification in the sampling design. Fourth, while there was not missing values observed for education in our population, we have missing values for several variables including CVD risk factors, employment and occupational social class. When compared with immigrants, natives were more likely to have missing values for all variables with the exception of cholesterol (Appendix 2). However, given the low percentage of missing values (<10%) relative to the analytical sample and the results presented in Tables 1–3, it is unlikely these exclusions have affected our results. Finally, given the limited sample size for the immigrant groups, it was not possible to examine length of stay in Spain by region of origin. Despite the small sample sizes and as a sensitivity analysis, we repeated the analyses presented in Table 3 accounting for length of stay among immigrants (Appendix 4). While we observed lower probabilities of at least three CVD risk factors among immigrants with at least 10 years of residence in Spain, the results were similar to the ones we presented in Table 3. Therefore, it is worth noting that acculturation, or the process by which foreign-born individuals adopt the culture and behaviors of the host country, may play a role [52]. Among the strengths of this study is the use of a large and representative sample of the Spanish adult population. The latter allows for the disaggregation by region of birth, as well as the examination of CVD clustering of risk factors and interactions of age, sex, and education with immigrant status.

## Conclusions

Our findings contribute to the study of inequalities in CVD risk factors, according to region of birth. The latter is crucial to identify populations at risk for CVD morbidity and mortality. In addition, the study examines the joint effects of immigrant status and educational, attainment which are important determinants of health inequalities. The findings show that the burden of CVD risk factors among immigrants is affected by education. Thus, our findings underscore the need to consider immigrant status and educational attainment in the design of prevention and health promotion strategies. These strategies should account for both the highest and the lowest educational attainment of immigrants, given their differential effect according to region of birth. Using such approach could reduce both the burden of CVD outcomes and their inequalities among immigrants.

## Supporting information

S1 Appendix (DOCX)
