# Supplementary material for: Cardiovascular disease risk factors in Spain: A comparison of native and immigrant populations
Source: PLoS One. 2020 Nov 30;15(11):e0242740. doi: 10.1371/journal.pone.0242740 (PMC7703989; doi:10.1371/journal.pone.0242740)
Supplement: S1 Appendix — (DOCX) [file pone.0242740.s001.docx]

Appendix 1. Countries included in each region of origin for the immigrant population Census Bureau, 2017

|  | **Eastern Europe** | **Latin America** | **Africa** |
| --- | --- | --- | --- |
| **Countries included** | Romania,  Bulgaria,  Ukraine,  Russia  Lithuania  Georgia  Armenia  Turkey  Serbia  Albania  Bosnia-Herzegovina  Croatia | Ecuador  Colombia  Venezuela  Peru  Bolivia  Dominican Republic  Cuba  Brazil  Paraguay  Uruguay  Honduras  Mexico  Nicaragua  El Salvador  Guatemala  Panama  Costa Rica  Dominica | Morocco  Senegal  Algeria  Nigeria  Equatorial Guinea  Mali  Gambia  Guinea  Mauritius  Cameroon  Guinea-Bissau  Egypt  Cape Verde  Ethiopia  South Africa  Ivory Coast  Angola  Republic of the Congo  Kenia  Burkina Faso  Sierra Leone  Liberia  Togo  Benin |

Appendix 2. Distribution of excluded cases and missing values for participants aged 25 to 64 years by nativity status, Spanish National Health Survey 2017

|  | **Natives**  **% (n=918)** | **Immigrants***  **%(n=566)** | **Total**  **%(n=1,484)** |
| --- | --- | --- | --- |
| Human Development Index >0.80 | 0.0 (0) | 64.4 (363) | 31.1 (363) |
| High Cholesterol | 0.2 (3) | 0.3 (1) | 0.2(4) |
| Smoking | 1.3 (9) | 0.2 (1) | 0.8 (10) |
| Overweight/obesity | 30.0 (303) | 8.8 (52) | 19.8 (355) |
| Unhealthy diet | 49.8 (429) | 7.9 (50) | 29.4 (479) |
| Insufficient physical activity | 1.9 (14) | 0.0 (0) | 1.0 (14) |
| Social Class | 13.6 (122) | 4.6 (32) | 9.3 (154) |
| Employment status | 2.3 (21) | 0.0 (0) | 1.2 (21) |
| Living arrangement | 0.9(17) | 0.2 (2) | 0.6 (19) |
| Asia | 0.0 (0) | 13.6 (65) | 6.6 (65) |

*P-value for Chi-squared statistics <0.001

Appendix 3. Prevalence Ratios and their 95% Confidence Intervals for region of origin on cardiovascular disease (CVD) risk factors, Spanish National Health Survey 2017.

| ***CVD risk-factors*** |  | **Unadjusted**  **PR (95% CI)** | **Adjusted***  **PR (95% CI)** |
| --- | --- | --- | --- |
| ***Hypertension*** | **Spain**  **Eastern** Europe  Latin America  Africa | 1.00  0.76 (0.52-1.10)  0.65 (0.49-0.85)  0.57 (0.37-0.87) | 1.00  1.17 (0.81-1.71)  0.94 (0.72-1.23)  0.80 (0.53-1.20) |
| ***High cholesterol level*** | **Spain**  **Eastern** Europe  Latin America  Africa | 1.00  0.79 (0.56-1.12)  0.78 (0.61-1.00)  0.61 (0.42-0.89) | 1.00  1.20 (0.84-1.71)  1.10 (0.87-1.40)  0.91 (0.64-1.28) |
| ***Diabetes*** | **Spain**  **Eastern** Europe  Latin America  Africa | 1.00  0.53 (0.24-1.19)  0.93 (0.61-1.42)  1.23 (0.69-2.19) | 1.00  0.88 (0.39-1.97)  1.47 (0.99-2.19)  1.70 (0.98-2.94) |
| ***Smoking*** | Spain  **Eastern** Europe  Latin America  Africa | 1.00  1.08 (0.98-1.19)  0.54 (0.47-0.62)  0.50 (0.41-0.60) | 1.00  1.11 (1.01-1.23)  0.56 (0.50-0.64)  0.50 (0.41-0.59) |
| ***Overweight/obesity*** | **Spain**  **Eastern** Europe  Latin America  Africa | 1.00  1.07 (0.95-1.20)  1.07 (0.98-1.16)  0.94 (0.83-1.07) | 1.00  1.16 (1.04-1.30)  1.19 (1.10-1.29)  0.93 (0.82-1.05) |
| ***Unhealthy diet*** | **Spain**  **Eastern** Europe  Latin America  Africa | 1.00  1.09 (1.04-1.14)  1.03 (0.99-1.07)  0.87 (0.80-0.95) | 1.00  1.05 (0.99-1.10)  1.00 (0.96-1.04)  0.82 (0.76-0.89) |
| ***Insufficient physical activity*** | **Spain**  **Eastern** Europe  Latin America  Africa | 1.00  1.17 (1.09-1.26)  1.02 (0.96-1.09)  1.26 (1.19-1.34) | 1.00  1.18 (1.09-1.26)  1.02 (0.95-1.09)  1.24 (1.17-1.31) |

*Adjusted for age (continuous), sex, employment status, living arrangement, education attainment and social class

In the unadjusted model, the probability of hypertension, smoking, cholesterol and unhealthy diet were lower in immigrants from Africa compared with Spanish natives (Appendix 3). These probabilities were lower for hypertension and smoking for immigrants of Latin America. When compared with natives, immigrants from **Eastern** Europe were more likely to be smokers (PR: 1.11; 95% CI: 1.01, 1.23), overweight/obese (PR: 1.16; 95% CI: 1.04, 1.30), and physically inactive (PR: 1.18; 95% CI: 1.09, 1.26) after adjustment. Immigrants from Africa (PR: 0.50; 95% CI: 0.41, 0.59) and Latin America (PR: 0.56; 95% CI: 0.50, 0.64) were less likely to smoke whereas those from Latin America more likely to be overweight/obese (PR: 1.19; 95% CI: 1.10, 1.29) when compared with their natives counterparts.

Appendix 4. Prevalence Ratios and their 95% Confidence Intervals for region of origin on cardiovascular disease risk factors by length of stay, Spanish National Health Survey 2017.

|  | **Unadjusted** | **Adjusted *** |
| --- | --- | --- |
| Spain  Eastern Europe ≤10 years  Eastern Europe >10 years  Latin America ≤10 years  Latin America >10 years  Africa ≤10 years  Africa >10 years | 1.00  0.98 (0.83-1.16)  1.29 (1.19-1.40)  0.76 (0.65-0.90)  0.95 (0.86-1.04)  0.56 (0.41-0.76)  0.90 (0.79-1.03) | 1.00  1.12 (0.95-1.31)  1.33 (1.23-1.45)  0.91 (0.78-1.06)  0.96 (0.88-1.06)  0.62 (0.45-0.84)  0.85 (0.74-0.97) |

CVD, cardiovascular disease

*Adjusted for age (continuous), sex, employment status, living arrangement, education attainment and

**occupational** social class,
